# Missed opportunity for tuberculosis case detection in household contacts in a high burden setting

**Published:** 2012-05-13

**Authors:** Juliet Nabbuye Sekandi, Hassard Sempeera, Justin List, Christopher Whalen

**Affiliations:** 1 Makerere University, School of Public Health, Kampala, Uganda; 2 Makerere University-Case Western Reserve University Research Collaboration, Kampala, Uganda; 3 Loyola University Chicago-Stritch School of Medicine, Maywood, IL, USA; 4 College of Public Health, University of Georgia, Athens, GA, USA

**Keywords:** Tuberculosis, Contact investigation, Active case finding

## Abstract

Contact investigation remains an essential component of tuberculosis (TB) control, yet missed opportunities to trace, medically examine, and treat close contacts of newly diagnosed index TB cases persist. We report a new case of active TB in a 21 year-old woman who was a household contact of a known TB index case in Kampala, Uganda. She was identified during a house-to-house TB case finding survey using chronic cough (≥2 weeks). This case study re-emphasizes two important public health issues in relation to TB control in developing countries; the need to promote active contact investigations by National TB programs and the potential complementary role of active case finding in minimizing delays in TB detection especially in high burden settings like Uganda.

## Introduction

Contact investigation is recommended by the Centers for Disease Control and Prevention as a strategy to identify, examine, and evaluate all persons who are at risk of infection with tuberculosis (TB) due to recent exposure to a diagnosed or suspected index case (1). This approach helps detect possible secondary active TB cases and those with latent TB infection that resulting from such exposure in order to initiate appropriate treatment and this strategy has been widely used in developed countries to stem the TB epidemic [1, 2]. In Uganda, the National TB Control Program employs passive case finding as the standard strategy for detection of index TB cases who present to health facilities but there is no surveillance system to follow up close or household contacts [3]. In the absence of such a system, community-based active case finding may provide an opportunity to fill the gap in detecting undiagnosed TB cases. A study done in Kampala, Uganda among household contacts of index TB cases identified 6% secondary cases, majority detected during baseline evaluations with the highest risk among children and HIV infected persons [4].

## Patient and case report

A 21 year-old university student was found to have active TB in May 2009 during a house-to-house TB case finding survey based on chronic cough (=2 weeks). After consenting to participate, she reported a history of productive cough associated with chest pain for 3 months without hemoptysis. She also reported occasional fevers, loss of appetite, weight loss, and general body weakness for about one month. She had no history of any other chronic diseases and no history of smoking or drinking alcohol.

The woman had been in close contact with a confirmed index TB case in her family. The index case experienced five months of coughing prior to diagnosis and started anti-TB chemotherapy in July 2008. The household comprised of five other family members: three adults and two children (nine and two years old). The woman also had regular contact with about 100 students in her class at the university. During the three months she experienced TB symptoms she attended full time classes spending about six hours in class for four to five days each week.

The woman used herbal medicine given to her by a traditional healer in the first month of onset of symptoms without much relief. A few weeks later, she sought care from a private provider in a drug store where she received anti-malarial and pain treatment with only mild relief of symptoms. No laboratory investigations were performed to confirm her diagnosis. At the time she was identified by the community active case finding survey team, she noticed her chest symptoms worsening but was not planning to seek further care until she started her university holiday recess.

### Investigation and treatment

During the survey, two sputum samples were collected, a tuberculin skin test (TST) was placed, and a rapid HIV test with same day results was performed. Smear microscopy using the Ziehl Neelsen acid-fast bacilli staining method and culture were performed. The woman was HIV negative, TST skin test positive at 18.6 mm after 48 hours, both sputum smears were positive (grade 3 + ), and sputum culture positive. After confirmation of active TB, the patient was started on standard TB treatment that she completed successfully.

## Discussion

This case study highlights four important issues that may contribute to effective TB control. First, crucial missed opportunities to investigate close contacts of index TB cases continue to occur. Second, patient delays in seeking appropriate healthcare for symptoms associated with TB persist. Third, inadequate health system coordination in evaluating potential TB suspects contribute to delayed treatment initiation. Fourth, community-based active case finding approaches that screen symptomatic individuals may help in curtailing transmission of TB by earlier identification of undiagnosed cases, shorten duration of infectiousness and reduce morbidity thus mitigating the impact of TB disease.

In Uganda, contact investigations are not currently a part of the national TB control policy, although it has been done in some research settings [4, 5]. The adaptation and implementation of the contact investigation policy remains difficult in low-income countries because of weak health systems, with limited health personnel and low healthcare budgets [3, 6]. Ideally in this case, at the time the index TB case was identified by the health system, household investigations should have been instituted. This could have perhaps led to initiation of TB preventive therapy, aversion of the secondary case of TB that we report, and any additional transmission by her.

The woman we present delayed to seek appropriate care for about 3 months from onset of symptoms. This is similar to the average patient delay of 4 months that was observed in index cases attending a referral TB clinic in urban Uganda [7]. The health system delays reflected in this case study arose from the patient having initially visited a herbalist and a drug store. This informal health system in Uganda often lacks the ability to perform adequate diagnostic evaluations for TB and other diseases. In order to reduce these delays, community-based active TB case finding, patient and health worker education should be reinforced as a supplemental strategy [8].

Studies in high-burden countries have shown that active case finding identifies additional active TB cases relatively early and could potentially reduce further spread of TB infection within households and communities [9, 10]. A study in Kampala that investigated 1,206 household contacts of 302 index TB cases showed that active TB occurred in 6% of the contacts, though molecular techniques were not used to confirm source transmission between household members. Patients less than 5 years old were particularly vulnerable and had increased risk for active TB [4, 5]. The household contacts of our reported case were evaluated, offered health education, and referred to the public health system for appropriate medical evaluation.

## Conclusion

This case report re-emphasizes two important public health issues in relation to TB control in developing countries: there is need to implement a policy to actively investigate close contacts of index TB cases who present for treatment within the health system in Uganda; and community-based active case finding approaches could play a supplementary role in identifying undiagnosed active TB cases that may delay to voluntarily seek care from the public system.
